# ﻿*Dryopterisjinpingensis*, a critically endangered diploid new species of Dryopteridaceae from Yunnan, China

**DOI:** 10.3897/phytokeys.239.118655

**Published:** 2024-03-19

**Authors:** Zheng-Yu Zuo, Jin-Mei Lu, Cun-Fu Li, De-Zhu Li

**Affiliations:** 1 Germplasm Bank of Wild Species, and Yunnan Key Laboratory of Crop Wild Relatives Omics, Kunming Institute of Botany, Chinese Academy of Sciences, Kunming, Yunnan, 650201, China Kunming Institute of Botany, Chinese Academy of Sciences Kunming China

**Keywords:** *Dryopteris* sect. *Diclisodon*, molecular phylogeny, new species, sexual diploid

## Abstract

*Dryopterisjinpingensis*, a new species of diploid, sexually reproductive ferns of Dryopteridaceae from Yunnan, southwestern China, is described and illustrated. Morphologically, *D.jinpingensis* is similar to *D.gaoligongensis* but unique in elongated lanceolate laminae, sessile or subsessile pinna stalks, and overlapping membranous scales adnate to stipe base. Phylogenetic analyses based on both plastome and the nuclear *AK1* gene sequences showed that *D.jinpingensis* is sister to *D.gaoligongensis*. A detailed taxonomic description with line drawings is provided, and its conservation status is evaluated to be critically endangered.

## ﻿Introduction

*Dryopteris* Adans. (Dryopteridaceae) is one of the largest fern genera in the world, comprising approximately 350–400 species (Wu et al. 2013; Sessa et al. 2015; PPG I 2016). It exhibits a wide distribution across temperate region and tropical montane areas, with a primary center of diversity in eastern Asia (Fraser-Jenkins 1986; Sessa et al. 2015). In China, there are some 167 species classified into four subgenera (Wu et al. 2013). Within the genus, Dryopterissect.Diclisodon (T. Moore) C. Chr. is of particular interest, as recent studies have revealed that many taxa in this section look similar in general appearance to their closely related known species, but actually exhibit significant difference in molecular data and close-up morphologies (Zuo et al. 2022a, b).

During our field work in Yunnan Province, China, we discovered a morphologically unique population of *Dryopteris* in Jinping County. The population exhibits a great similarity with *D.gaoligongensis* Z.Y. Zuo, Jin Mei Lu & D.Z. Li we described earlier (Zuo et al. 2022b). Through extensive morphological and phylogenetic studies, we have confirmed that this population represents a distinct species and accordingly we propose a new species within D.sect.Diclisodon.

## ﻿Methods

Living plants of the potentially new taxon and *D.gaoligongensis* were observed in the field to conduct morphological comparisons. In particular, the scales, frond shape, and pinna stalks, were observed and counted. Additionally, specimens or photographs of specimens of related species of D.sect.Diclisodon deposited in the herbaria CSH, K, KUN, MICH, PE, and PYU were also examined.

Ploidy levels were estimated using flow cytometry (BD FACSCalibur, U.S.A.) by measuring nuclear DNA content (2C value) of young fresh leaves, with *Zeamays* L. (1C = 2.70 pg) (Bennett and Smith 1976) as reference standard. Reproductive mode was estimated by counting the spores in each sporangium, with 64 spores and 32 spores per sporangium representing sexual and apogamous reproduction, respectively (Walker 1979). At least ten intact sporangia were observed under a mini-microscopy (Yuantu 100×, China).

A young leaf of the potentially new taxon was collected from living plants in the field, and total genomic DNA was extracted from 30 mg of silica-gel dried leaf material using the modified 4× CTAB DNA extraction method (Doyle and Doyle 1987). For plastid genome (plastome), library preparation and Illumina sequencing were conducted at the Germplasm Bank of Wild Species, Kunming Institute of Botany (CAS). *De-novo* assemblies, connecting and annotation were constructed using GetOrganelle v.1.7.0 (Jin et al. 2020), Bandage 0.8.1 (Wick et al. 2015) and Geneious 9.1.4 (Kearse et al. 2012), respectively, based on the previously published plastome of *Dryopterisgaoligongensis* (NC_067598). The new obtained plastomes were uploaded to NCBI (Suppl. material 1).

The products of PCR amplification of the low-copy nuclear *AK1* gene (*AK4F*: 5′-GATGAAGCCATCAAGAAACCA-3′; *AKR2*: 5′-ATGGATCCAGCGACCAGTAA-3′) (Hori et al. 2021) were cloned and sequenced at Tsingke Biotechnology Co., Ltd. Kunming, with at least six colonies for each sample (Suppl. material 2).

Two matrices were constructed for phylogenetic analyses. We extracted nine identical plastid regions from two newly obtained plastomes of the potentially new taxon, and added them to our previous combined plastid matrix (including 57 samples, Suppl. material 1, 3) (Zuo et al. 2022a, b). The second matrix consisted of 50 nuclear *AK1* sequences, two of which were newly obtained and the others from previous studies (Hori et al. 2021; Zuo et al. 2022a, b; Wei et al. 2024). The two matrices were aligned and corrected using MAFFT v.7.017 (Katoh et al. 2002) and Geneious 9.1.4 (Kearse et al. 2012), respectively.

We used Bayesian inference (BI) and Maximum likelihood (ML) analyses to infer the phylogenetic relationships. The BI analysis was performed using MrBayes 3.2.6 (Ronquist et al. 2012) in four Markov chain Monte Carlo (MCMC) chains in parallel, with ten million generations and one tree sampled every 1000 generations. The first 25% of trees were discarded as burn-in. The ML analyses were conducted using IQ-TREE 1.6.12 (Nguyen et al. 2015) with the GTR+R6 model and 2000 ultrafast bootstrap replicates.

## ﻿Results

**Figure 1.**
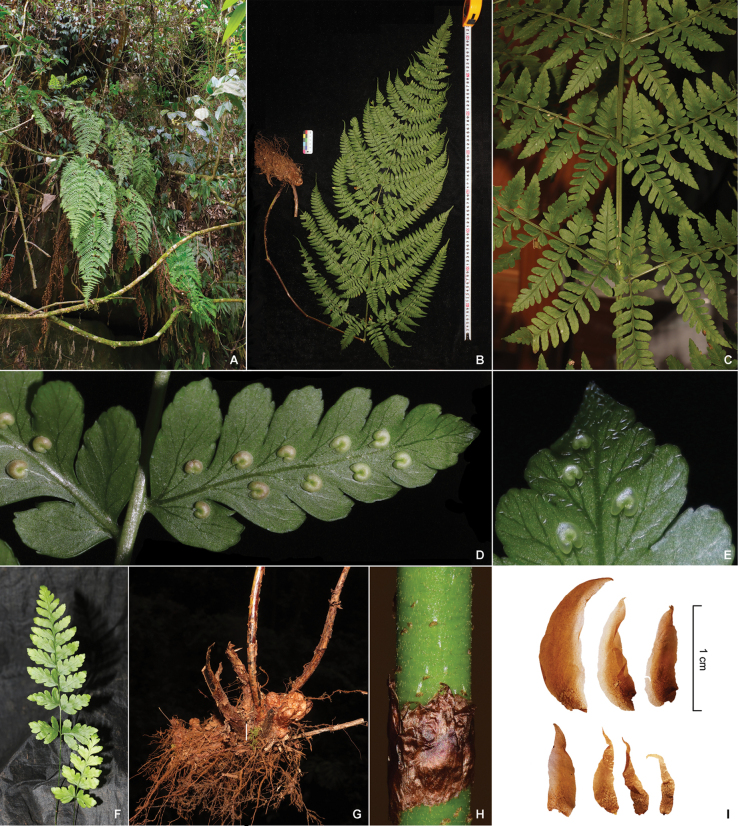
Photos of *Dryopterisjinpingensis* (*Z.Y. Zuo 5378*) **A** habitat **B** plant **C** proximal part of the lamina **D** sori on ultimate pinnules **E** glandular hairs on the abaxial surface of pinnules **F** young plant **G** rhizome **H** portion of stipe base, showing adnate scale **I** scales of stipe.

**Figure 2.**
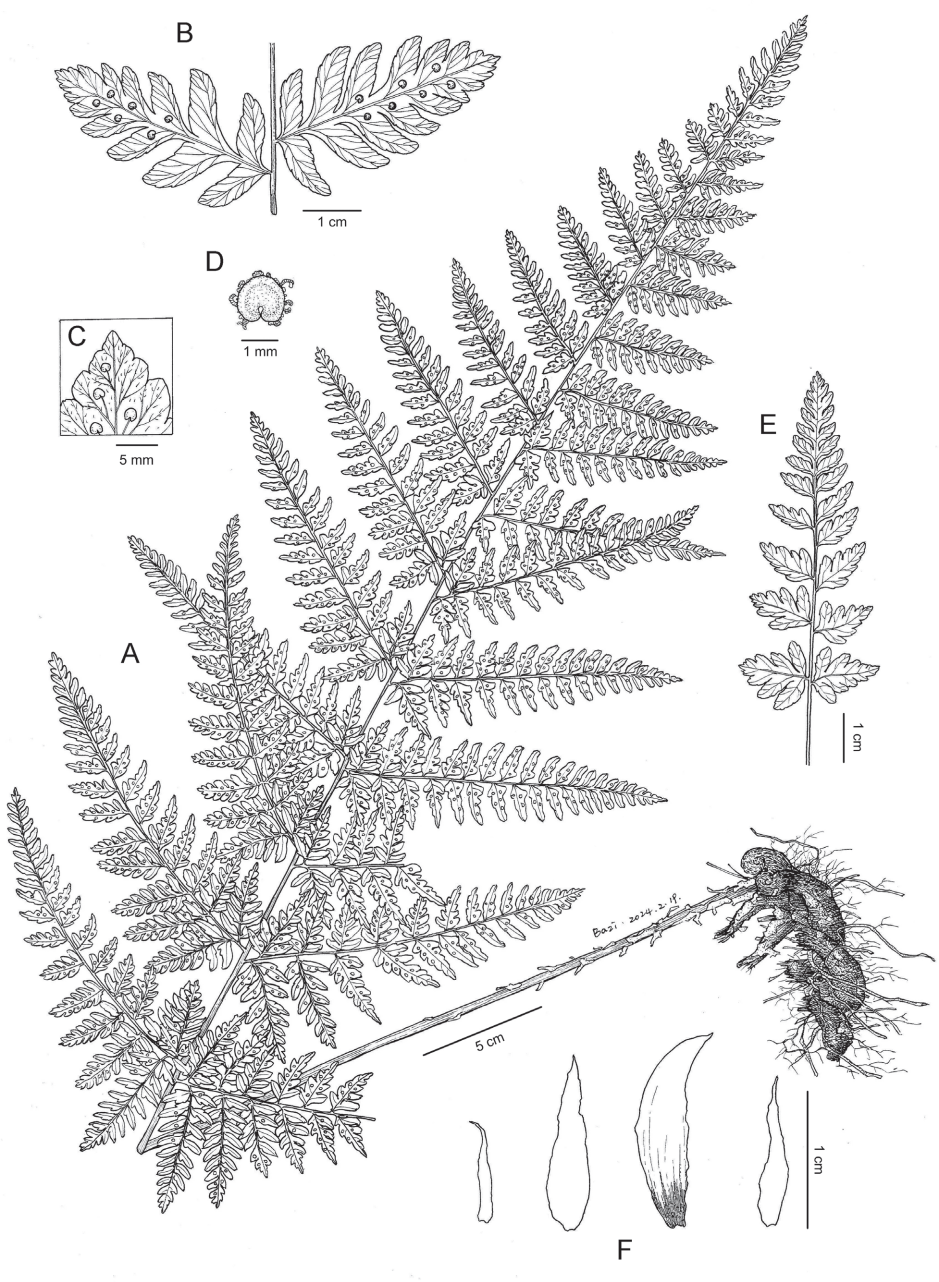
Illustration of *Dryopterisjinpingensis* Z.Y. Zuo, Jin Mei Lu & D.Z. Li **A** plant **B** sori on ultimate pinnules **C** glandular hairs on the abaxial surface of pinnules **D** indusia **E** young plant **F** scales of stipe (Drawn by Yi-Fan Li, based on *Z.Y. Zuo 5378*).

**Figure 3.**
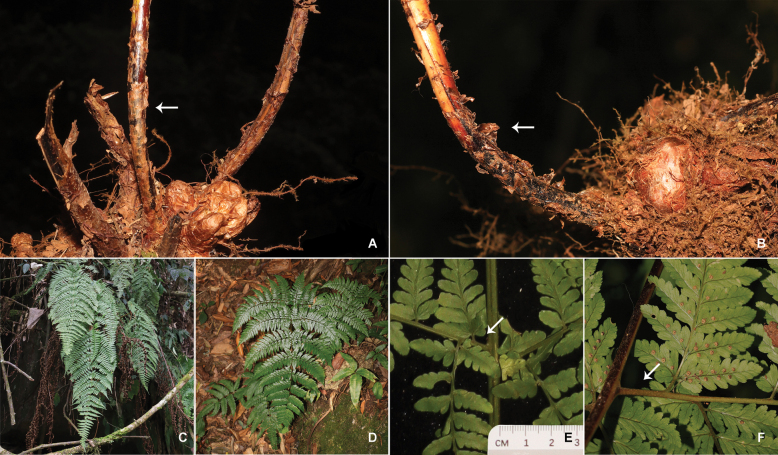
Morphological comparison of *Dryopterisjinpingensis* (**A, C, E**) and *D.gaoligongensis* (**B, D, F**) **A, B** rhizome and stipe base **C, D** lamina **E, F** pinna stalks of basiscopic pinnules.

The morphological comparison revealed that the potentially new taxon exhibited a great similarity to *D.gaoligongensis* in overall morphology. Both species have stout and creeping rhizomes, 3- to 4- large pinnate fronds, and largest and longest basal basiscopic pinnule. However, there are some distinct differences. The scales of *D.gaoligongensis* are brown, ovate-lanceolate, and entire, while the potentially new taxon has thin membranous and lanceolate scales. More importantly, most scales of the potentially new taxon are overlapping and adnate to the stipe base. The fronds of *D.gaoligongensis* are deltate-lanceolate to ovate-lanceolate, and basal pinnae with stalk up to 5 cm. In contrast, the potentially new taxon has elongated lanceolate fronds, and pinna stalks of the basal pinnae are sessile or subsessile (less than 1 cm). In addition, the potentially new taxon was found on cliffs of the valley in forests (Figs 1–3, Table 1).

**Table 1.** Diagnostic characteristics comparison between *Dryopterisjinpingensis* and *D.gaoligongensis*.

Characters	*Dryopterisjinpingensis*	*D.gaoligongensis*
Scales on stipe base	Thin membranous, lanceolate, mostly overlapping, adnate to stipe	Thick membranous, ovate-lanceolate, mostly scattered, not adnate to stipe
Lamina	Elongated lanceolate	Deltate-lanceolate to ovate-lanceolate
Pinna stalks	Sessile or subsessile, less than 1 cm in the basial pairs of pinnae	Stalked, 2–5 cm in the basal pairs of pinnae
Habit	On the cliff of the valley in forests	On the ground in forest
Altitude	1000–1100 m	2200–2500 m

**Figure 4.**
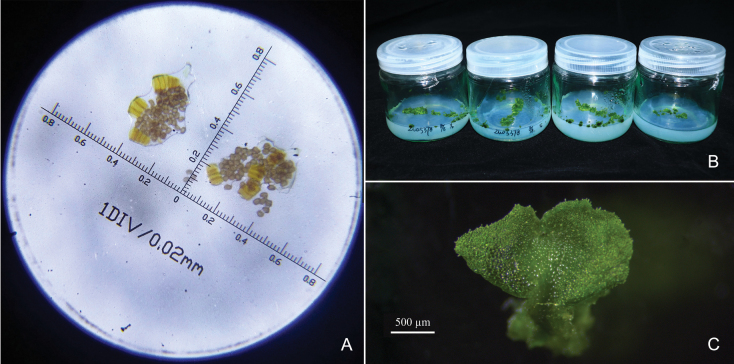
Spores and prothallus of *Dryopterisjinpingensis***A** 64 spores in one sporangium **B** cultured prothallus on 1/2 Murashige and Skoog plant cell culture medium (MS) **C** prothallus.

The DNA amount of three samples of *Zuo5378* (*Zuo5378-1* from one population, *Zuo5378-2* & *Zuo5378-3* from another nearby population) was estimated to be 15.2 ± 0.2 pg, which is very close to that of *D.gaoligongensis* (15.1 ± 0.4 pg) and other diploid species of D.sect.Diclisodon (e.g., *D.sabaei*, 14.2 ± 0.3 pg; *D.subexaltata*, 12.5 ± 0.3 pg; Hori et al. 2021). All spore counts showed that the potentially new taxon has 64 normal spores per sporangium, and the spores could germinate to produce prothallus (Fig. 4). The results of flow cytometry and spore counting implied that the potentially new taxon is a diploid, sexually reproductive species.

**Figure 5.**
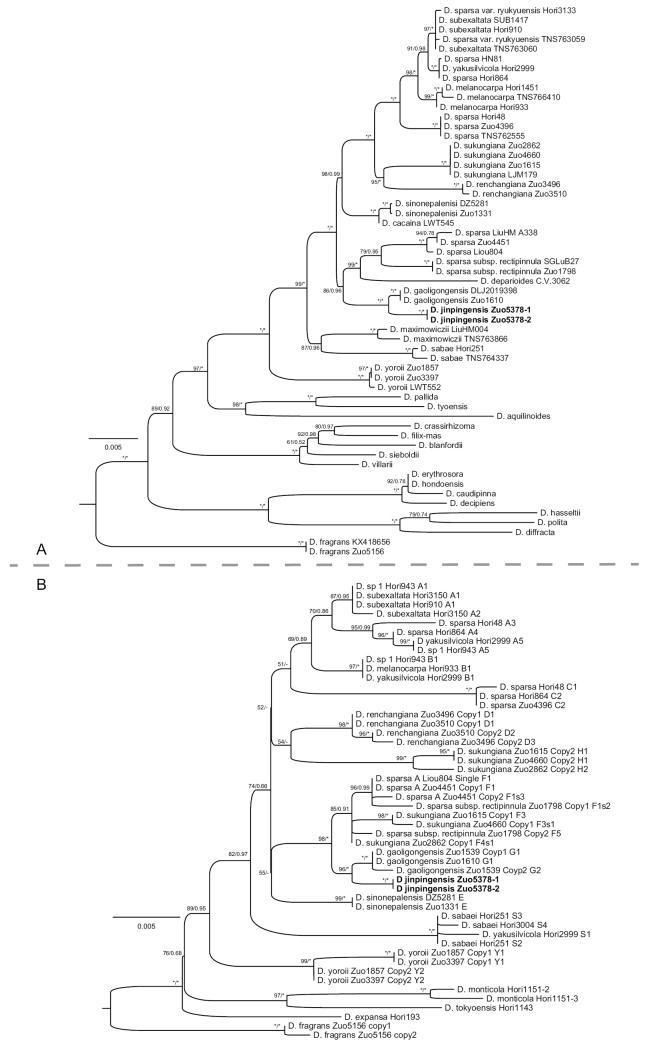
Maximum likelihood phylograms of Dryopterissect.Diclisodon based on nine plastid regions (**A**) and nuclear gene *AK1* (**B**). ML ultrafast bootstrap support values (UFBS) and the posterior probabilities of Bayesian inference (BIPP) are indicated near nodes (UFBS/BIPP). The stars (*) indicate UFBS=100% or BIPP=1.00, the minus (-) indicate UFBS<50% or BIPP<0.50. The name of the new species is in bold.

Phylogenetic analyses of both the plastome data (Fig. 5A) and nuclear *AK1* data (Fig. 5B) concordantly revealed that the potentially new taxon is sister to *D.gaoligongensis*. Only one haplotype of nuclear *AK1* was found in the potentially new taxon.

## ﻿Discussion

Morphological comparison and phylogenetic analyses show that the potentially new taxon is a member of D.sect.Diclisodon, and is closely related to *D.gaoligongensis*. They are not only similar in overall morphology, but also in nuclear DNA content, ploidy level, and reproductive mode. However, phylogenetic data and some significant morphological differences (Table 1) suggest that they are two distinct species that may have a common ancestor but have undergone different speciation in different environments. *Dryopterisgaoligongensis* exclusively grows on the ground of the Gaoligong Mountains at an elevation of 2200–2500 meters, while the newly discovered species grows on the cliffs inside the valley at an altitude of 1000–1100 meters. We speculate that environmental differences and isolation have resulted in a lack of gene flow between the two species, which ultimately led to their differentiation and speciation.

### ﻿Taxonomic treatment

#### ﻿ Dryopteris jinpingensis

Taxon classificationPlantaePolypodialesDryopteridaceae

Z.Y.Zuo, Jin Mei Lu & D.Z.Li sp. nov.

77C0E297-2C02-5E7A-9B32-282838D7A7A5

urn:lsid:ipni.org:names:77338637-1

Figs 1 , 2 in Chinese: 金平鳞毛蕨 (jīn píng lín máo jué)

##### Type.

China. Yunnan: Jinping, 25°17′N, 98°46′E, alt. 1050 m, 20 April 2023, *Z.Y. Zuo 5378* (Holotype, mounted in 3 cross-referenced sheets, KUN-1585758! Isotype: KUN-1585759!).

##### Diagnosis.

*Dryopterisjinpingensis* is similar to *D.gaoligongensis* with stout and creeping rhizome, 3- to 4-pinnate large frond, and largest and longest basal basiscopic pinnule. However, *D.jinpingensis* differs from it in the elongated lanceolate lamina, sessile or subsessile pinna stalks (less than 1 cm), and overlapping membranous and thin scales adnate to stipe base.

##### Description.

***Plants*** 70–140 cm tall. ***Rhizome*** stout, creeping, up to 20 cm long and 4 cm in diameter, densely clothed with brown, lanceolate, entire scales. ***Fronds*** approximate, stipe shorter than lamina, ca. 30–60 cm, brown at base, upper stramineous, densely scaly; scales thin, lanceolate, entire, brown, overlapping and adnate to the stipe base. ***Rachis and costae*** hairy when young, glabrous when mature. ***Lamina*** papery, not glossy, broadly elongated lanceolate, ca. 40–100 × 20–50 cm, 3- to 4-pinnate, base not narrowed, apex acuminate, abaxial with glandular hairs when young, glabrous when mature. ***Pinnae*** 15–30 pairs, opposite, oblique, sessile or subsessile (less than 1cm). Pinnae lanceolate, basal pinnae largest, deltoid-lanceolate, up to 40 × 18 cm, apex caudate-acuminate. ***Pinnules*** 25–35 pairs, opposite at base and alternate upward, lanceolate, base broadly cuneate, usually asymmetrical, apex long acuminate; basal basiscopic pinnule largest and longest, ca. 12 × 4 cm, base widest, 2-pinnate; Segments oblong, apices obtuse and spinulose, margin shallowly lobed to several serrate. ***Veins*** pinnate, forked, distinct on both surfaces. ***Sori*** close to costa on pinnules; indusia orbicular-reniform, entire. Reproductive mode and ploidy level: diploid sexual.

##### Distribution and habitat.

Presently only known from Jinping County, Yunnan Province, southwestern China, with two documented small populations near each other. It grows on the cliff of the valley in subtropical evergreen broad-leaved forests, at an altitude of 1000–1100 meters.

##### Etymology.

The specific epithet “jinpingensis” refers to its type locality, Jinping County, in the border between south Yunnan of China and Vietnam.

##### Conservation status.

*Dryopterisjinpingensis* should be classified as critically endangered (CR) according to the IUCN guidelines (IUCN Standards and Petitions Committee 2022), due to its narrow distribution with only two small populations with fewer than 50 plants. In order to conserve and save this rare and endangered species, we have begun to propagate it using the *in vitro* culture from spores, facilitated by the Germplasm Bank of Wild Species.

## Supplementary Material

XML Treatment for Dryopteris jinpingensis

## ﻿Additional information

### Author ORCIDs

Zheng-Yu Zuo https://orcid.org/0000-0002-8334-6132

Jin-Mei Lu https://orcid.org/0000-0002-8728-8750

Cun-Fu Li https://orcid.org/0009-0006-1286-9928

De-Zhu Li https://orcid.org/0000-0002-4990-724X

### Data availability

All of the data that support the findings of this study are available in the main text or Supplementary Information.

## Supplementary materials

Supplementary material 1 The voucher information and GenBank accession numbers of complete plastid genome used in this study Data type xlsx https://binary.pensoft.net/file/1005525This dataset is made available under the Open Database License (http://opendatacommons.org/licenses/odbl/1.0/). The Open Database License (ODbL) is a license agreement intended to allow users to freely share, modify, and use this Dataset while maintaining this same freedom for others, provided that the original source and author(s) are credited.Zheng-Yu Zuo, Jin-Mei Lu, Cun-Fu Li, De-Zhu Li

Supplementary material 2 The voucher information and GenBank accession numbers of nuclear marker *AK1* used in this study Data type xlsx https://binary.pensoft.net/file/1005526This dataset is made available under the Open Database License (http://opendatacommons.org/licenses/odbl/1.0/). The Open Database License (ODbL) is a license agreement intended to allow users to freely share, modify, and use this Dataset while maintaining this same freedom for others, provided that the original source and author(s) are credited.Zheng-Yu Zuo, Jin-Mei Lu, Cun-Fu Li, De-Zhu Li

Supplementary material 3 The voucher information and GenBank accession numbers of plastid regions used in this study Data type xlsx https://binary.pensoft.net/file/1005527This dataset is made available under the Open Database License (http://opendatacommons.org/licenses/odbl/1.0/). The Open Database License (ODbL) is a license agreement intended to allow users to freely share, modify, and use this Dataset while maintaining this same freedom for others, provided that the original source and author(s) are credited.Zheng-Yu Zuo, Jin-Mei Lu, Cun-Fu Li, De-Zhu Li
